# 

**Published:** 1871-05

**Authors:** 


Vol. XII. MAY, 1871. No. 5.

### CHICAGO Medical Examiner,

N. S. DAVIS, M.D., EDITOR,

AND

F. H. DAVIS, M.D., ASSIST.-EDITOR.

TERMS, $3.00 PER ANNUM, IN ADVANCE.

CHICAGO:

ROBERT FERGUS' SONS, PRINTERS,

12 CLARK AND 242—248 ILLINOIS STREET

1871.

